# The efficiency of adjusting nutrient solution renewal frequency on physicochemical properties and microbial community of cucumber exudates under closed cultivation tank

**DOI:** 10.1371/journal.pone.0298910

**Published:** 2024-08-16

**Authors:** Yaqing Gao, Jiajun Sang, Hao Liang, Yanhai Ji, Mingchi Liu

**Affiliations:** 1 Vegetable Institute of Beijing Academy of Agriculture and Forestry Sciences, Beijing, China; 2 North China Key Laboratory of Urban Agriculture, Ministry of Agriculture, Beijing, China; Universidade Federal de Minas Gerais, BRAZIL

## Abstract

The closed nutrient solution management method allows for the recycling and utilization of nutrient solutions, improving the efficiency of water and fertilizer utilization. This study was conducted to investigate the effects of changing the frequency of nutrient solution renewal and method of nutrient supply on the microbial communities composition, yield, and quality in closed soilless systems by using high-throughput sequencing technology and combining the physicochemical properties of root exudate solution. The results showed that different nutrient solution management modes had a significant impact on the structure and diversity of root exudate solution microbial communities. The abundance and diversity of microorganisms in inorganic perlites were correlative with EC. The abundance and diversity of bacterial communities in the root exudate solution of open liquid supply (CK) were higher than that of closed liquid supply, while the abundance and diversity of fungal communities in the root exudate solution of closed liquid supply (T1, T2, T3) were higher than that of open liquid supply. As the frequency of nutrient solution interval decreased, the accumulation of salt in root exudate solution and the richness and diversity of the fungal community also decreased, especially increasing the K^+^, Ca^2+^, and Mg^2+^ contents, which were positively correlated with potential beneficial *Candidatus_Xiphinematobacter*, *Arachidicoccus*, *Cellvibrio*, *Mucilaginibacter*, *Taibaiella* communities and decreasing the content of soluble protein, Vitamin C content, but not significantly increased cucumber yield.

## 1. Introduction

Perlite culture was one of the most widely used soilless culture types with the advantages of water saving, fertilizer saving, and low cost. With the development of facility agriculture, the application of perlite had also increased, but most perlite cultivation adopted an open nutrient management mode [1, 2] and showed that 20-80% of the excess nutrient solution under the open nutrient management mode was drained, resulting in a waste of water and fertilizer resources. In contrast, in the closed-loop soilless culture system, most of the collected drainage was reused, improving water and fertilizer efficiency, saving costs, and protecting the environment [3].

Microbe plays an important part in soilless culture perlite [4]. The exudation released from plant roots into the perlite was the main food source of the microbe and also the driving force of microbial population density and activity [5, 6]. In the process of microbe metabolism, nitrification and ammoniation provided good nutrient conditions for plants and accelerated the circulation of nutrient elements in the micro-ecosystem [7]. The main function of microbe in soilless culture was to participate in nutrient transformation and improve plant stress resistance [8]. In cultivating muskmelon with different perlites, Cho et al. [9] showed that the number of microbes was soil > perlite > nutrient solution, and bacteria was the most abundant in rhizosphere microbe. The microbe in the perlite was easily affected by environmental factors, such as EC, pH, different fertilization systems, etc [10]. Schwarz et al. [11] showed that the number of nitrifying bacteria and ammonifying bacteria in rock wool and the nutrient solution decreased with increasing EC. A shift in pH toward acidity can lead to a shift in the microbial decomposer community from bacteria to fungi [12].

The microbe research mainly concentrated on soil, however, little was known about the microbial community in the root exudate solution of plants under soilless culture. Therefore, in this experiment, a closed inorganic matrix circulating pot cultivation system was used to explore the influence on the changes of the microbial community in the root exudate solution of cucumber, the EC of root exudate solution, cucumber yield and quality by changing the frequency of nutrient solution interval and method of supplying the nutrient solution, in order to implement a closed nutrient solution management mode for cucumber cultivation under the perlite cultivation system.

## 2. Materials and methods

### 2.1. Plant husbandry and experimental design

The experiment was carried out in the glass greenhouse of the Vegetable Research Institute of Beijing Academy of Agriculture and Forestry Sciences from January to May 2021(116°29’E, 39°94’N). The "Jingyan 118" variety cucumber (Beijing Jingyan Yinong Seed Technology Co., Ltd) was selected as the test material. The cultivation system adopted closed inorganic perlite pot culture with nutrient solution recycling developed by the Vegetable Research Institute of Beijing Academy of Agriculture and Forestry Sciences (patent No.: ZL201510214349. X; For details, see S1 Fig, the cultivation slot length × width × height = 48 × 20 × 13 cm). The nutrient solution in the closed-loop system was pumped from independent supply tanks through a drip irrigation system, and the excess nutrient solution entered the independent supply tanks while in the opened-system return, the liquid was directly discharged into the sewer pipe.

The volume of each independent supply tank was 200 L and cucumber seedlings was recycled for 10 days on average. The nutrient solution used for irrigation was composed of N, P, K, Ca, Mg, and S elements in the formula with content of 11.60, 1.30, 6.51, 2.24, 1.00, and 1.39 mmol∙L^-1^, respectively. Each treatment had 22 cultivation pots, 2 plants of each cultivation pot, and the plant spacing was 20 cm, with a row spacing of 40 cm and 150 cm processing spacing each. Four weeks after germination, seedlings with four true leaves that were uniform in size were selected and transplanted to a cultivation pot full of perlite. After 14 days, different test treatments were started. The experiment consisted of 5 treatments: CK: replace nutrients and open supply nutrient solution after use; T1: 3 d replaceed a nutrient solution and a closed supply nutrient solution; T2: 5 d replaced a nutrient solution and a closed nutrient solution; T3: 7 d replaced the nutrient solution and a closed nutrient solution; T4: replaced nutrient and a closed nutrient solution supply after use. Each process had 3 replicates. After the preparation of the nutrient solution, no renutrient solution was added until it was renewed. The EC in the nutrient solution pool was measured every two days. A pump connected to drip emitters controlled by a timer was used for irrigation. Each plant was irrigated with nutrient solution 6 times during the period from 07: 30 to 17: 30, each time for 8 minutes, with an interval of 120 minutes, and the total irrigation was 2 L.

### 2.2. Sampling and analysis methods

#### 2.2.1. Quality index

The quality of cucumber was determined during the harvest period, and each treatment was repeated at least 3 biological times. The soluble sugar content in cucumber was measured using the anthrone colourimetric method [13], and the vitamin C (Vc) content was analyzed by the 2,6-dichlorophenol titration method [14]. The soluble protein content was measured using the coomassie brilliant blue [15]. The digital sugar meter (ATAGO PAL-BX/ACID1) was used to analyze the total soluble solids (TSS). Used a ruler to measure the vertical diameter, handle the length of the fruit, and used a vernier caliper to measure the horizontal diameter of the fruit.

#### 2.2.2. Sampling of root zone microbe

Before pulling the seedlings, root-zone solution (150 ml) was collected with a root solution extractor (Model: KH05-KH100R, Beijing Haifida Technology Co., ltd) installed between the plants and stored in the refrigerator at -80°C to determine the microbial community’s diversity.

#### 2.2.3. Physicochemical analysis of the root area solution

During the experiment, every 6 days, 150 ml of root exudate solution was collected with a root solution extractor for each treatment and stored in a refrigerator at 4°C to determine the EC value. During the seedling stage (84 d after planting), the mineral elements in root exudate solution, including total nitrogen, total phosphorus, potassium ions, calcium ions, and magnesium ions, were determined by Perkin Elmer, Optima 8000, USA.

#### 2.2.4. Microbial community analysis

150 mL of root exudate solution samples in different treatments at 84 days was filtered through a sterile membrane filter (0.22 μm) and then transferred into a new tube. Genomic DNA was extracted from 150 mL of root exudate solution samples using the TGuide S96 Magnetic Soil DNA Kit [Tiangen Biotech (Beijing) Co., Ltd.] according to the manufacturer’s instructions. The extracted DNA was evaluated using 1.8% agarose gel, and the concentration and quality were determined using a Nano-Drop ND-2000 spectrophotometer (Nano-Drop, Wilmington, DE, USA). For the root exudate solution community analyses, the PCR primers 338F (5´-ACTCCTACGGGAGGCAGCA-3´)- 806R (5´-GGACTACHVGGGTWTCTAAT-3´) [16] and ITS1F (5´-CTTGGTCATTTAGAGGAAGTAA-3´) - ITS2 (5´-GCTGCGTTCTTCATCGATGC-3´) [17] were used to amplify the bacterial 16S rRNA gene and the fungal internal transcribed spacer (ITS), respectively. The PCRs and product purification were performed as described by [18]. PCR productions were subjected to high-throughput sequencing by Shanghai Personal Biotechnology Co., Ltd. (Shanghai, China), using the Illumina MiSeq sequencing platform (Illumina, Inc., CA, United States). All sequences were first conducted with the QIIME package (Quantitative Insights Into Microbial Ecology) (v 0.33) for Illumina sequencing data through quality filtering and chimera removal, and the retained effective tags were used to perform operational taxonomic unit (OTU) and species annotation. The unique sequence was classified into OTUs under the threshold of 97% identity using USEARCH (version 10.0). Chimeric sequences were identified and removed using Uchime (version 8.1). The taxonomy of each 16S rRNA and ITS gene sequence was analyzed by UCLUST against the Silva (Release132, http://www.arb-silva.de) and UNITE (Release 8.0, https://unite.ut.ee/)) database using a confidence threshold of 90%.

#### 2.2.5. Statistical analysis

Analysis of variance (ANOVA) was carried out for the alpha diversity and the relative abundance of microbial and mineral elements characteristics by using SPSS software version 20.0. Canoco 5.0 was used for principal component analysis (PCA) and redundancy analysis (RDA). The mineral nutrient element content, cucumber yield, and quality were processed by Microsoft Excel 2019 software. Data were expressed as means with standard deviation (SD). Means were separated by Fisher’s least significant differences (LSD) tests at a significant level α = 0.05. The classification method combined sequence alignment with a naive Bayesian classifier, and the clustering method used similarity classification (sequence similarity was 0.97). Redundancy analysis (RDA) of microbial and environmental factors was conducted with Canoco 5 software, and the difference was significant (*P* < 0.05) [19, 20].

## 3. Results

### 3.1. Variation characteristics of electrical conductivity of cucumber root exudate solution

**Fig 1:**
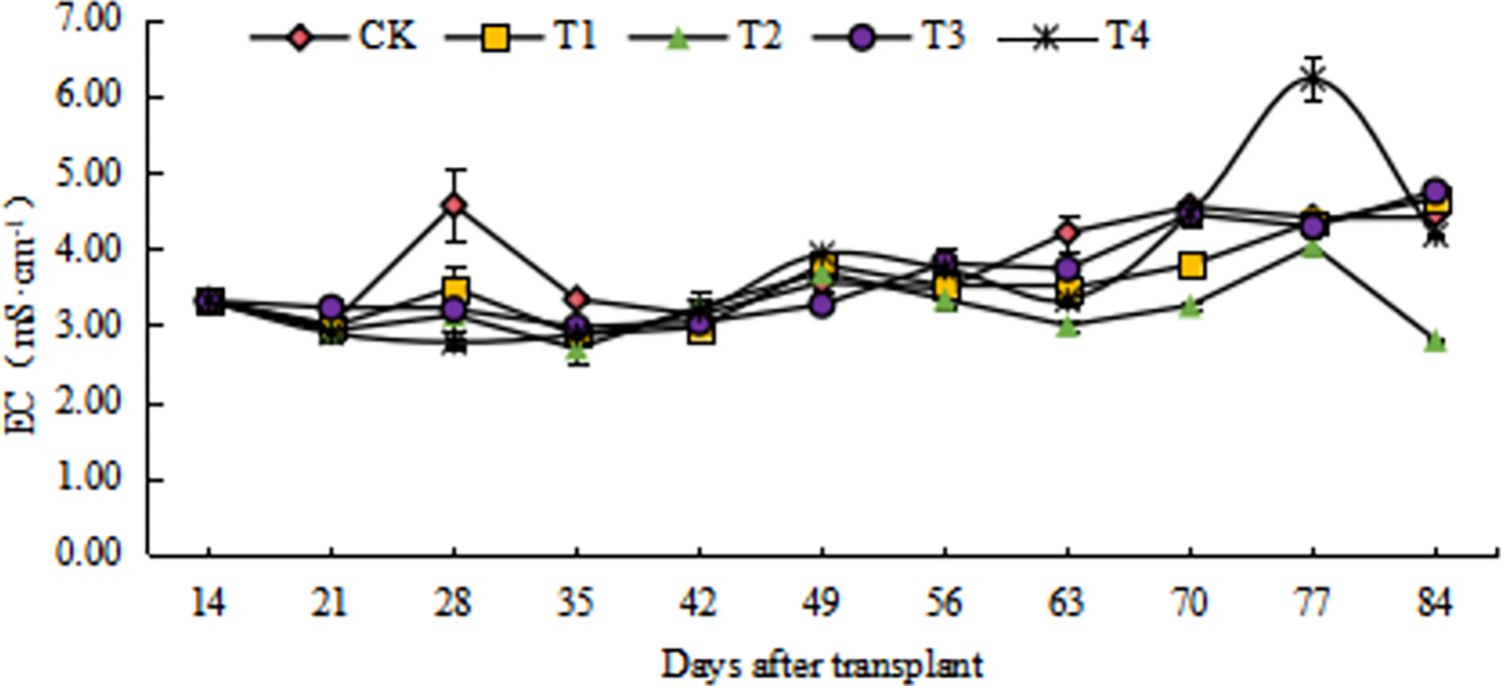
Change of EC with different nutrient solution management modes in the root exudate solution of cucumber.

The EC of root exudate solution increased with the growth period during the whole growth period. The EC of root exudate solution under CK and T4 was increased, and the highest EC reached 4.56 mS·cm^-1^ and 6.21 mS·cm^-1^, respectively (Fig 1). At first, from 14 d to 21 d, EC showed a downward trend, then, a slow process of rising to fall from 21 d to 35 d. Planting for 35 d (early melon stage), the EC showed CK > T1 > T3 > T2 > T4. At this time, the EC of open management was significantly higher than that of closed management. From 35 d to 56 d, except T3, there was a process from slow rise to slow decline. Planting for 56 d (peak fruiting stage), the EC showed T3 > T4 > T1 > CK > T2, at this time, the closed management was gradually higher than the open management (CK) and reached a significant difference level (*P <* 0.05). The EC of all treatments rose slowly from 56 to 70 d. On 70 d to 84 d, T1 and T4 had a similar parabola process, while other treatments had no significant difference. Planting for 84 d (before seedling pulling), the EC showed T3 > T1 > CK > T4 > T2. Compared with that before planting, the EC of CK, T1, T3, and T4 treatment increased by 34.14%, 40.18%, 43.20%, and 25.98% respectively, while T2 treatment decreased by 15.41%.

### 3.2. Analysis of mineral nutrient elements in cucumber root exudate solution

**Fig 2:**
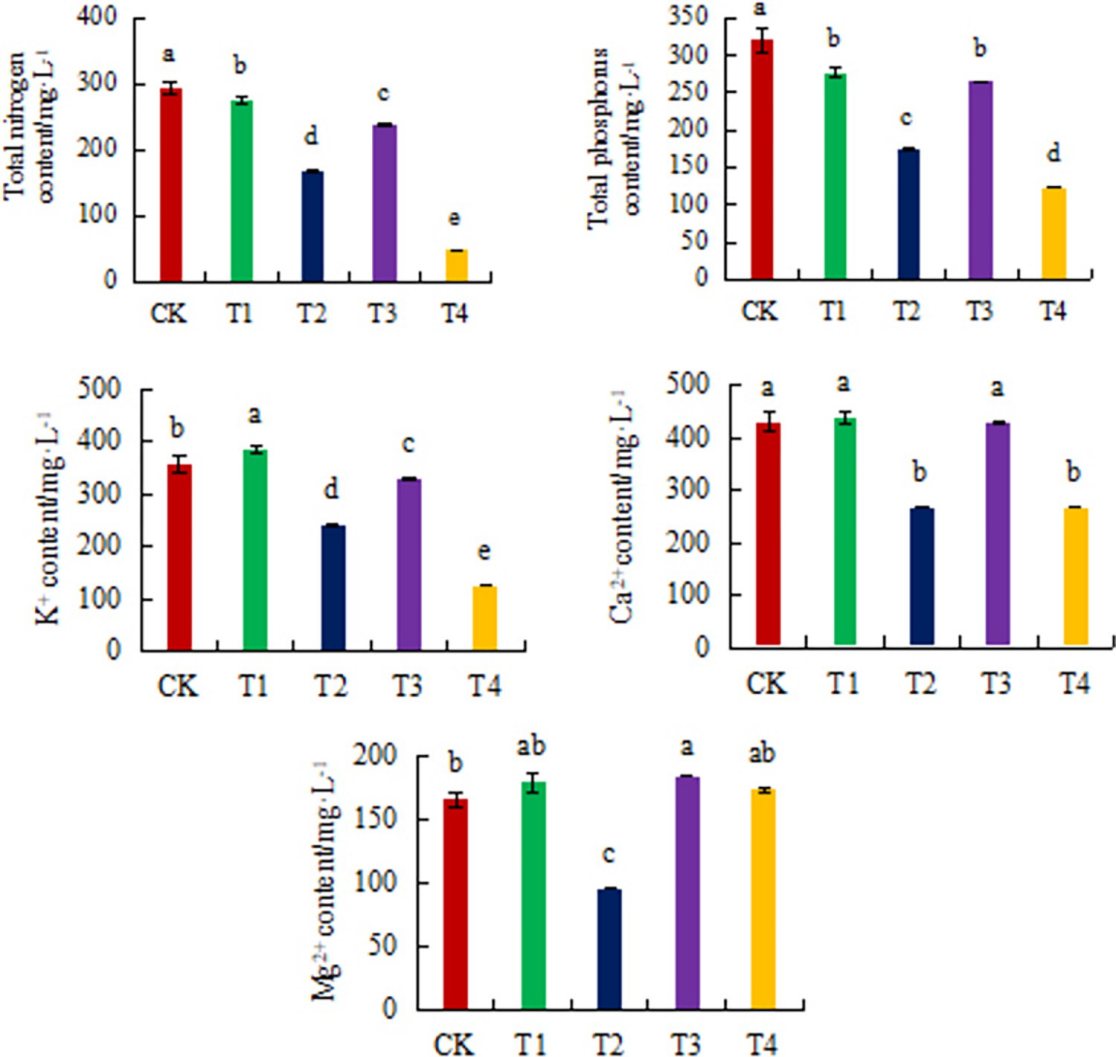
Change of mineral nutrients with different nutrient solution management modes in the root exudate solution of cucumber.

The order of mass contribution rate of various nutrient content to the accumulation of salt in the root zone was Ca^2+^, K^+^, total phosphorus (TP), total nitrogen (TN), Mg^2+^ (Fig 2). By 84 days after planting (before pulling seedlings), the TP and TN content showed CK > T1 > T3 > T2 > T4, K^+^ and Ca^2+^ content showed T1 > CK > T3 > T2 > T4, Mg^2+^ content showed T3 > T1 > T4 > CK > T2. The open management mode (CK) was conducive to the accumulation of TN, TP, and Ca^2+^content in the root zone, which was not conducive to Mg^2+^ accumulation.

### 3.3 The microbial community composition in cucumber root exudate solution

#### 3.3.1 Alpha diversities of bacterial and fungal communities in the root exudate solution of cucumber

The OTU was the artificial classification unit which was utilized in the phylogenetics and population genetics studies. All sequences was divided OTUs based on different levels of similarity, and each OTU corresponded to a representative sequence. Totals of 1,143,530/1141337 reads and 8597/4159 OTUs were obtained from the genetic sequencing of the bacterial and fungal microbial communities, respectively, in the 15 root exudate solution samples obtained from the treatments. (Table 1). The order of bacterial OTUs was T1 < T4 < T2 < CK < T3. Compared with T1, the OTUs of CK, T2, T3, and T4 increased by 20.50%, 16.50%, 21.48% (*P <* 0.05), and 6.37%, respectively. The order of fungi OTU was T1 < CK < T3 < T4 < T2. The fungi OTU in CK, T2, T3, and T4 treatments had no significant difference, while T2 treatment was significantly increased by 57.38% than T1 treatment (*P <* 0.05). According to Chao1, ACE and phylogenetic distance (PD) _whole_tree index showed that T3 treatment had the highest bacterial abundance, while T1 had the lowest. The fungi richness in root exudate solution of CK treatment was the highest, and T1 was the lowest. According to Shannon and Simpson indexes, the bacterial diversity in root exudate solution of T2 treatment was the highest, while that of T1 was the lowest. The diversity of fungi in root exudate solution was no significant difference among treatments.

**Table 1:** Diversity index and estimated sample coverage of root exudate solution samples.

treatment	OTUs	Coverage (%)	PD_whole_tree	Richness		Diversity	
				Chao1	Ace	Shannon	Simpson
Bacterial							
CK	961 ± 54 ab	99.79	67.02 ± 1.93 bc	1136 ± 42 a	1118 ± 58 ab	7.80 ± 0.29 a	0.9845 ± 0.0031 a
T1	764 ± 39 c	99.79	58.75 ± 2.14 d	904 ± 46 b	878 ± 53 b	4.94 ± 0.22 c	0.8534 ± 0.0416 b
T2	915 ± 72 ab	99.80	68.65 ± 0.89 ab	1064 ± 77 ab	1050 ± 31 ab	8.26 ± 0.08 a	0.9898 ± 0.0007 a
T3	973 ± 12 a	99.73	71.83 ± 0.42 a	1180 ± 33 a	1136 ± 36 ab	6.95 ± 0.26 b	0.9644 ± 0.0019 a
T4	816 ± 11 bc	99.75	63.50 ± 1.17 c	1094 ± 87 ab	1185 ± 174 a	7.92 ± 0.18 a	0.9890 ± 0.0031 a
Fungi							
CK	263 ± 4 ab	99.94	64.77 ± 1.82 a	513 ± 66 a	702 ± 97 a	5.62 ± 0.32 a	0.9266 ± 0.0196 a
T1	244 ± 3 b	99.95	59.71 ± 2.54 a	387 ± 49 a	482 ± 81 a	4.91 ± 0.93 a	0.8240 ± 0.1235 a
T2	384 ± 19 a	99.94	80.82 ± 4.58 a	454 ± 36 a	573 ± 110 a	6.60 ± 0.71 a	0.9556 ± 0.0310 a
T3	268 ± 5 ab	99.95	64.18 ± 0.71 a	377 ± 20 a	468 ± 40 a	5.45 ± 0.43 a	0.8848 ± 0.0499 a
T4	320 ± 82 ab	99.95	68.93 ± 12.60 a	489 ± 54 a	545 ± 47 a	5.90 ± 1.31 a	0.8887 ± 0.0976 a

OTUs: operational taxonomic units; Chao1: the richness estimator; Ace: the abundance-based coverage estimator; Shannon: shannon diversity index; Simpson: simpson diversity index. Different letters indicated significant differences among treatments according to Duncan’s multiple range test at *p <* 0.05 level. The same as below. *Data given in the form mean ± standard errors

#### 3.3.2 Analysis of bacterial and fungal community of cucumber root exudate solution

**Fig 3:**
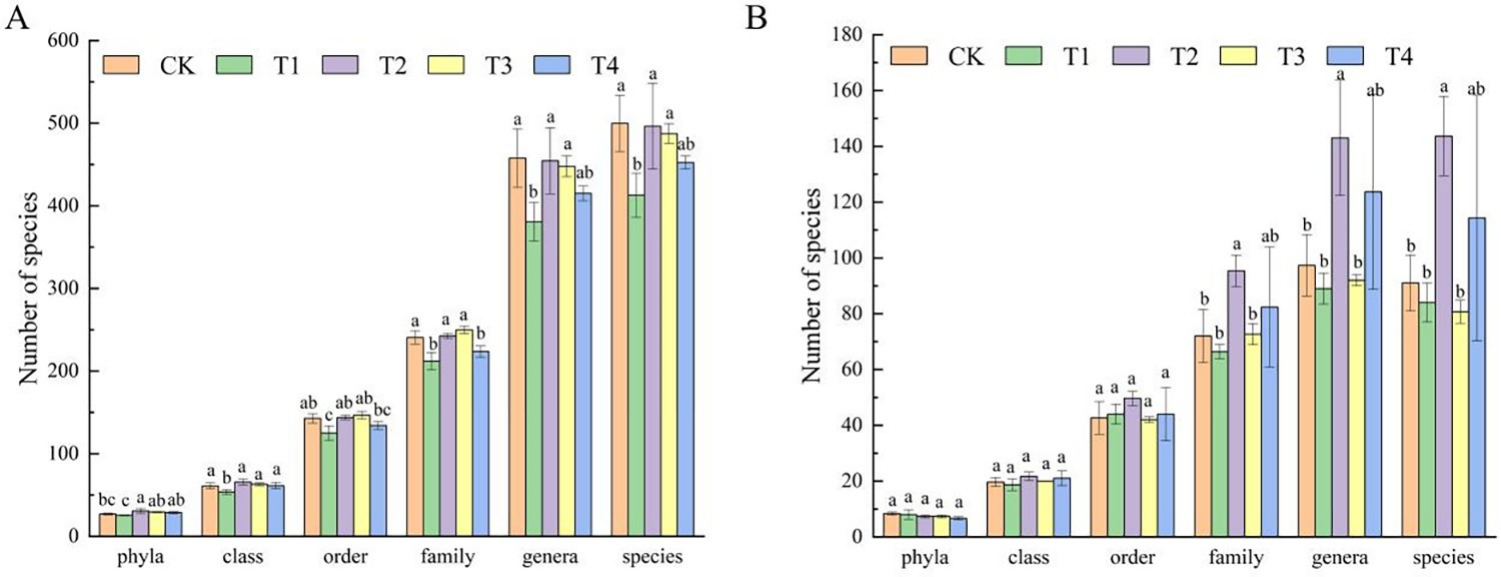
Number of bacterial (A) and fungal (B) species annotated by different classification levels with different nutrient solution management modes.

The bacteria in the root exudate solution included 39 phyla, 91 classes, 212 orders, 373 families, 744 genera, and 824 species (Fig 3A). Different nutrient management models led to differences in species numbers at different taxonomic levels. Among them, the species number of CK, T2, and T3 was significantly higher than T1 treatment. The fungi in the root exudate solution include 10 phyla, 35 classes, 84 orders, 175 families, 319 genera, and 369 species (Fig 3B). The number of species under different nutrient solution management modes has no significant difference from the phyla to order. From the family, the number of species increased. At the genus classification level, the number of species in each treatment reached the maximum, and the number in T2 treatment was significantly higher than that in CK, T1, and T3 treatments.

**Fig 4:**
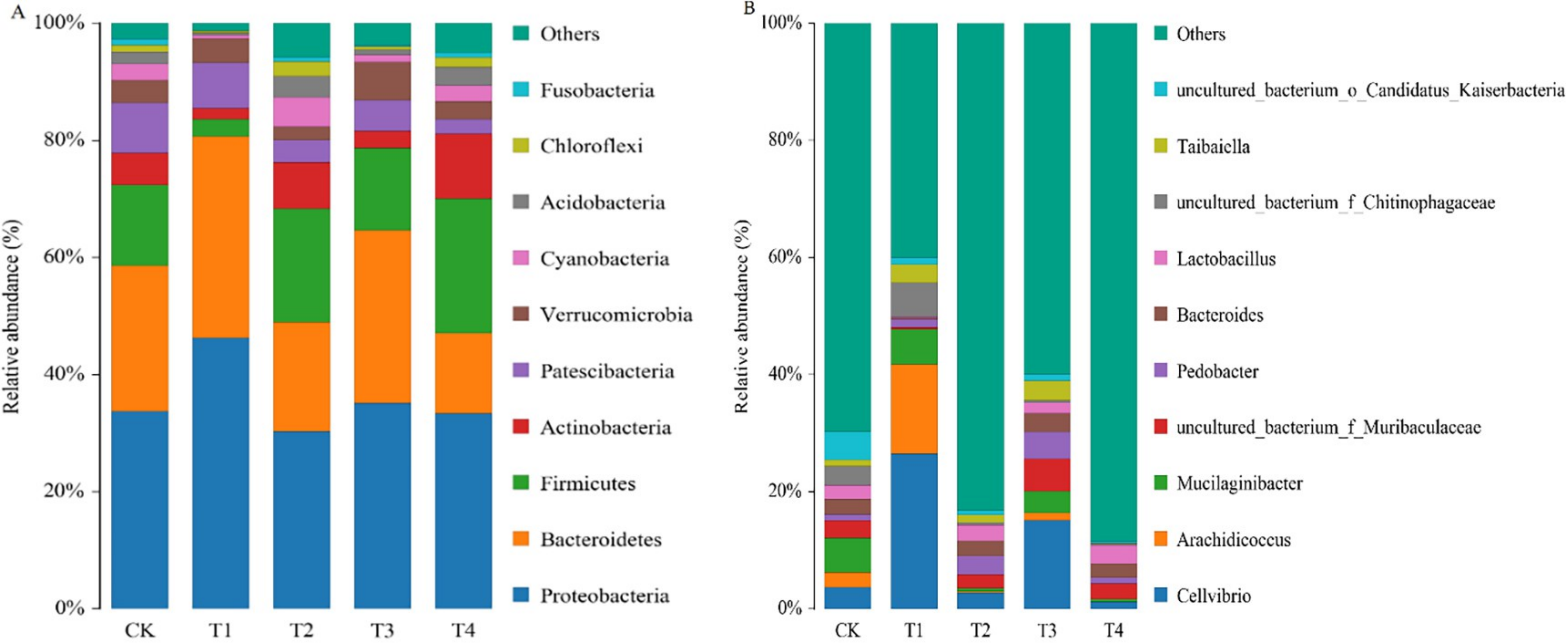
Changes of bacterial community composition at phyla (A) and genera (B) levels in different treatments.

**Fig 5:**
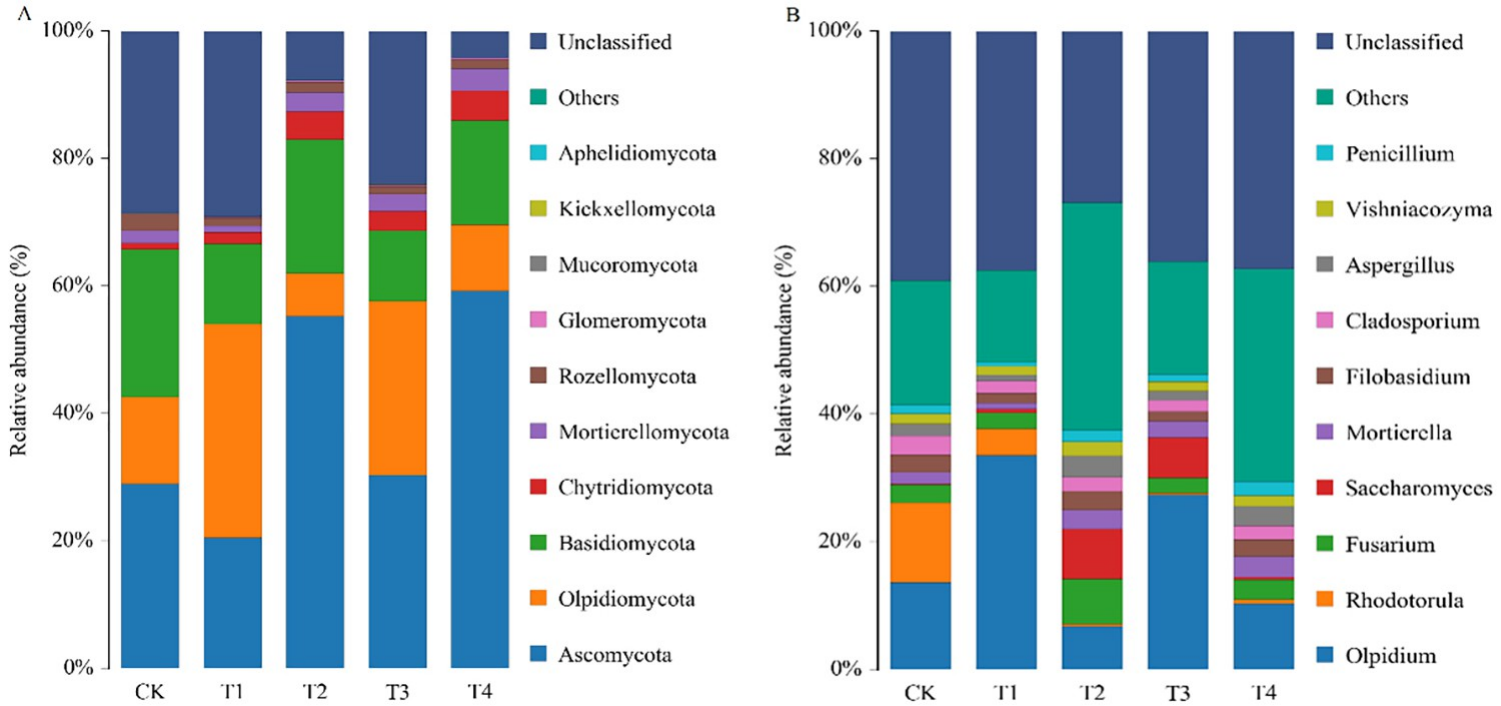
Changes of fungal community composition at phyla (A) and genera (B) levels in different treatments.

The dominant bacterial phyla were *Proteobacteria*, *Bacteroidetes*, *Firmicutes*, *Actinobacteria*, *Patescibacteria*, *Verrucomicrobia*, *Cyanobacteria*, *Acidobacteria*, *Chloroflexi*, *Fusobacteria* in the root exudate solution (Fig 4A). The relative abundance of *Proteobacteria* in treatments was accounting for 30.65-46.04%, compared with CK, that of T1 treatment was increased by 35.57% (*P <* 0.05). The relative abundance of *Bacteroides* was 13.77-34.65%, and the relative abundance of *Firmicutes* was 2.92-22.90%. The dominant bacterial genera in the root exudate solution were *Cellvibrio*, *Arachidicoccus*, *Mucilaginibacter* (Fig 4B). The dominant fungal were *Basidiomycota*, *Chytridiomycota*, *Mortierellomycota*, *Rozellomycota*, *Glomeromycota*, *Mucoromycota*, *Kickxellomycota*, *Aphelidiomycota* (Fig 5A). The dominant fungal phyla in the root exudate solution were *Ascomycota*, *Olpidiomycota*. The *Ascomycota* was the most abundant, accounting for 20.50-59.32%. The relative abundance of that was 28.85, 20.50, 56.11, 31.17, and 59.32% in five treatments, respectively. Compared with CK, the *Ascomycota* of T2, T3, and, T4 treatment was increased by 94.49% (*P <* 0.05), 8.04%, and 105.62% (*P <* 0.05), while T1 treatment decreased by 28.94%. The relative abundance of *Olpidiomycota* and *Basidiomycota* was 7.35%~33.53% and 11.13%~22.96%, respectively. The relative abundance of the top ten fungi in T1 was 48.07%, followed by 46.08% in T3, 41.30% in CK, 39.50% in T2, and 29.36% in T4. The dominant fungal genera in the root exudate solution were *Olpidium*, *Rhodotorula*, *Fusarium*, *Saccharomyces*, *Mortierella*, *Filobasidium*, *Cladosporium*, *Aspergillus*, *Vishniacozyma*, *Penicillium* (Fig 5B). The dominant genera of T1, T3, and T4 were *Olpidium*, the dominant genera of CK were *Olpidium* and *Rhodotorula*, and the dominant genera of T2 were *Olpidium* and *Fusarium*.

### 3.4 Analysis of microbial and environmental factors

**Fig 6:**
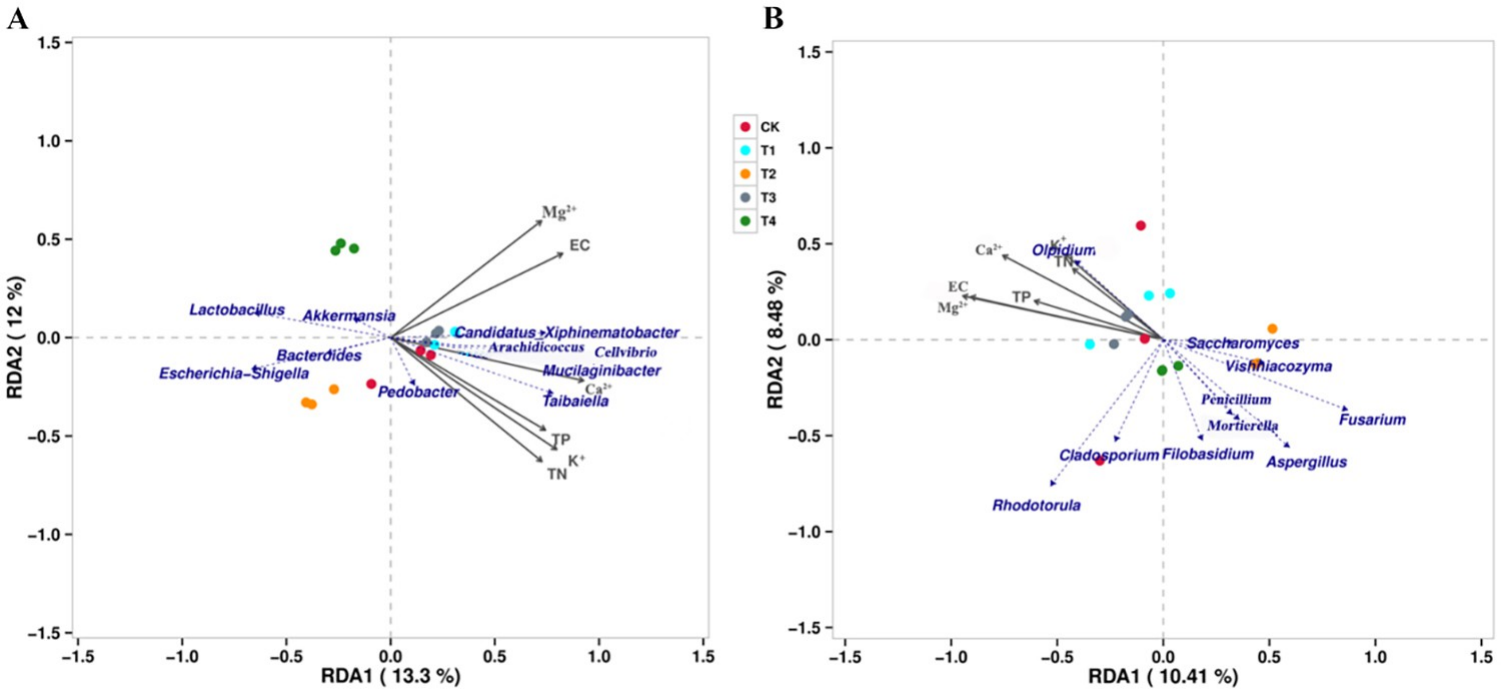
Redundancy analysis (RDA) based on the relative abundances of the top 20 bacterial (A) and fungal (B) genera of root exudate solution in different treatments.

The RDA analysis showed that the first and second RDA components explained 25.30% and 18.89% of the total bacterial and fungal variations, respectively (Fig 6). For bacteria, the first component (RDA 1), which explained 13.30% of the total variation, separated the different treatment. The C*andidatus_xiphinematobacter*, *Arachidicoccus*, *Cellvibrio*, *Mucilaginibacter*, *Taibaiella*, and *Pedobacter* were related to the Mg^2+^, K^+^, Ca^2+^, EC, TP, TN contents (Fig 6A). The bacterial genera in the T2 were dominated by *Bacteroides and Escherichia-shigella* (Fig 6A; Table 2). For fungi, the first component (RDA 1), which explained 10.41% of the total variation in fungal genera, separated the different treatments. Furthermore, the *Olpidium*, *Rhodotorula*, and *Cladosporium* were related to the Mg^2+^, EC, TP, Ca^2+^, K^+^, and TN contents (Fig 6B; Table 2).

**Table 2:** Redundancy analysis of root exudate solution microorganisms and environment factors.

Name	Explain	Pseudo-F	*P*
Bacteria			
N	44.4	10.4	0.002*
P	35.1	7.0	0.008*
K^+^	51.7	13.9	0.002*
Ca^2+^	45.2	10.7	0.006*
Mg^2+^	9.9	1.4	0.232
EC	17.0	2.7	0.094
Fungi			
N	7.0	1.0	0.364
P	8.0	1.1	0.334
K^+^	7.3	1.0	0.366
Ca^2+^	9.1	1.3	0.272
Mg^2+^	7.1	1.0	0.396
EC	7.5	1.1	0.350

### 3.5 The quality and yield of cucumber

Different solution management modes (CK: opened; T4: closed) had a significant impact on the nutritional quality of cucumbers (Table 3). Compared with CK, the soluble sugar content of T4 was increased by 25.14% (*P <* 0.05), while the soluble protein content was reduced by 15.28% (*P <* 0.05). There was no significant difference in vitamin C content among the treatments. Different nutrient solution intervals (T1: 3 d; T2: 5 d; T3: 7 d) had a significant impact on the nutritional quality of cucumber. The vitamin C content was the highest in T1, which was increased by 13.82, 13.52, and 21.92% compared with T2, T3, and T4, respectively. The soluble sugar content was high in T4, which was increased by 16.24 and 21.49% compared to T1 and T3, respectively. T3 has a high content of soluble protein, which was increased by 77.3 and 54.10% compared with T2 and T4, respectively. There were significant differences in the yield of cucumber in opened (CK) and closed (T4) solution management modes. The single plant yield of CK was 25.80% higher than that of T4. However, there was no significant difference in the interval solution management mode (T1:3 d; T2: 5 d; T3: 7 d).

**Table 3:** Effects of different nutrient solution management modes on the nutritional quality of cucumber.

Treatment	Vitamin C Content (mg·100 g^-1^)	Soluble sugar content (%)	Soluble protein content (mg·g^-1^)	Single plant Yield (kg)
CK	10.53±0.03 bc	3.66±0.03 b	0.72±0.11 bc	1.58 a
T1	12.85±0.37 a	3.94±0.03 b	0.91±0.04 ab	1.37 b
T2	11.29±0.10 b	4.49±0.22 a	0.53±0.05 c	1.33 b
T3	11.32±0.10 b	3.77±0.04 b	0.94±0.02 a	1.30 b
T4	10.54±0.34 bc	4.58±0.25 a	0.61±0.07 c	1.25 b

## 4. Discussion

### 4.1 Analysis of salt accumulation in cucumber root exudate solution under different nutrient management modes

Different nutrient solution management modes havd different salt accumulation in the cucumber root zone. In a previous study, Paprika (*Capsicum annuum* L.) was grown in the rockwool culture with a recycled nutrient solution, and the EC significantly increased from the initial set value of 2.5 dS∙m^-1^ for 14 to 42 days after treatment and then decreased [21]. Tomato crops in open and closed systems EC increased [22]. In this study, The EC value increased during the growing period under the open and closed solution modes, which was consistent with the study of tomato, but not completely consistent with the study of Paprika. It may be that the study of Paprika was supplemented with water and fresh nutrient solution for adjusting EC and constant volume 3 times a week, and this study was supplemented with water and fresh nutrient solution for adjusting EC every 3, 5, 7, or even longer (30 d). Among them, interval nutrient solution every 5 d reduced EC during the seedling pulling period, which was consistent with the study of Paprika. Therefore, under the closed soilless culture, proper renewal of nutrient replacement frequency can reduce the accumulation of salt in the root zone, which was conducive to the growth of plants.

### 4.2 Impacts of different nutrient solution management modes on mineral nutrients in cucumber root exudate solution

In a tomato study, ion (K^+^, Mg^2+^, Ca^2+^, SO4^2-^, Na^+^, and Cl^-^) and salinity were significantly accumulated when the nutrient solution was continuously circulated for 4 weeks [23]. In this study, also when pulling seedlings, the ion contribution rate of root exudate solution of each treatment from large to small was Ca^2+^, K^+^, TP, TN, and Mg^2+^. Compared with the open system, except for Mg^2+^, TN, TP, Ca^2+^and K^+^ in the closed system had decreased, which was consistent with the previous tomato study [23]. Similarly, a study of the Gerbera crop suggested supplying K^+^ and TN at higher K: (K^+^ Ca^+^ Mg^2+^) and NH4/TN ratios than those recommended for open systems [24]. In order to mitigate nutrient imbalances in the solution provided to the crop, it was imperative to appropriately replenish the drain solution [25, 26]. Therefore, in our research, compared to the open system, in order to avoid ion imbalances, it was recommended to replace the nutrient solution every 3 days to some extent.

### 4.3 Impacts of different nutrient solution management modes on microbial community, yield, and quality

The abundance and diversity of microbial groups in closed hydroponic systems were affected by multiple factors such as crop type, perlites, or irrigation water [27]. The community structure of bacteria and fungi in the rhizosphere of melon under 6 different nutrient treatments was analyzed. It was found that significant changes in the microbial community composition between growth stages. Bacterial communities were dominated by the following three phyla *Proteobacteria*, *Firmicutes*, *and Bacteroidetes*. By contrast, fungal communities were majorly dominated by *Ascomycota* and *Basidiomycota*. At the genus level, bacterial communities were dominated by *Brevibacillus* and *Lysobacter* were dominant at the fruiting and harvesting stages. Fungal taxa primarily consisted of *Trichoderma* and *Fusarium* [28]. In this study, bacterial communities were dominated by *Proteobacteria*, *Bacteroidetes*, *Firmicutes*, and fungal communities were majorly dominated by *Ascomycota*, *Olpidiomycota*, and *Basidiomycota*. At the genus level, bacterial communities were dominated by *Cellvibrio*, *Mucilaginibacter*, and fungal taxa primarily consisting of *Olpidium*, *Rhodotorula*, *Olpidium*, and *Fusarium*. This was similar to the classification of melon at the phyla level, but not completely the same at the genera level. Because the taxonomic diversity of fungi and bacteria was substantially affected by both abiotic and biotic variables and exhibited contrasting diversity patterns in response to environmental variables in various niches [29, 30].

Closed-cycle soilless systems had been recommended in commercial production because they can minimize water and fertilizer losses [31]. However, salt accumulation in the growth matrix was the recycling system’s main challenge. Open cycle systems were usually used to reduce the salinity level in the matrix. However, a closed circulation system can obtain higher water efficiency. Several cultural approaches had been suggested to mitigate salt stress in closed recirculating systems, such as cultivar screening and nutrient management [32]. In this study, Increasing the interval frequency of nutrient solution appropriately can reduce the salinity of root zone. High salinity levels of nutrient solution led to negatively impacted nutrient uptake, such as NO3-, K, P, and Ca^2+^, and shoot and root nutrient content [33]. In this study, open cycle systems increased unit yield, but reduced quality. On the contrary, the closed cycle soilless system was beneficial for improving quality, but a little reduced yield.

## 5. Conclusions

The closed cycle soilless systems can improve the cucumbers’ quality, increase the relative abundance of root potential beneficial microbial community, improve the root physicochemical properties, and decrease the number of pathogens. More specifically, closed cultivation (1) increased the soluble sugar and Vitamin C content and improved cucumber quality. (2) stimulated the relative abundance of biological community such as *Proteobacteria*, *Bacteroides*, *Cellvibrio*, *Arachidicoccus*, *Mucilaginibacter*, *Fusarium*, *and Olpidiomycota*, *Olpidium*, which will help to reduce the incidence of harmful cucumber diseases; and (3) increased the K^+^, Ca^2+^, Mg^2+^ contents, which were positively correlated with potential beneficial *Candidatus_Xiphinematobacter*, *Arachidicoccus*, *Cellvibrio*, *Mucilaginibacter*, *Taibaiella* communities and decreasing the EC content. In conclusion, the closed cycle soilless systems can improve cucumber quality, adjust the root’s microbial and physicochemical balance, alleviate root salinization, achieve water-fertilizer saving, and environmentally friendly development, promoting agricultural sustainability.

## Supporting information

S1 Fig The cultivation system adopts closed inorganic perlite pot culture with nutrient solution recycling developed by the Vegetable Research Institute of Beijing Academy of Agriculture and Forestry Sciences (patent No.: ZL201510214349. X; the cultivation slot length × width × height = 48 × 20 × 13 cm). (TIF)
