# Supplementary figures and images for: The efficiency of adjusting nutrient solution renewal frequency on physicochemical properties and microbial community of cucumber exudates under closed cultivation tank
Source: PLoS One. 2024 Aug 16;19(8):e0298910. doi: 10.1371/journal.pone.0298910 (PMC11329137; doi:10.1371/journal.pone.0298910)

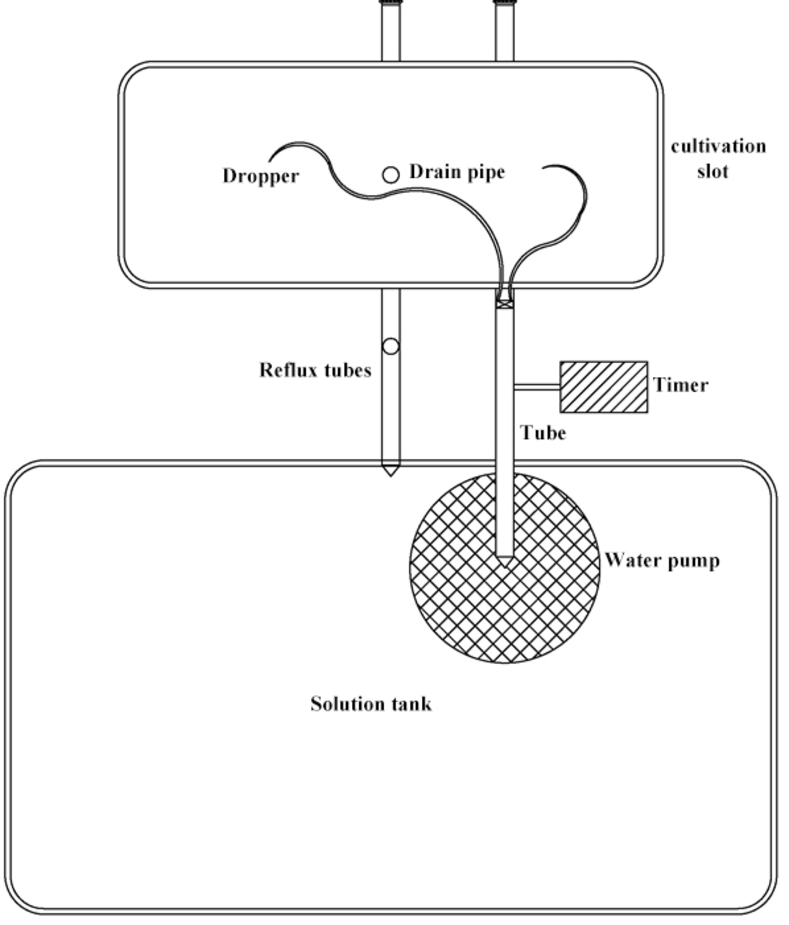

Supplement: S1 Fig — (TIF) [file pone.0298910.s001.tif]
